# Exosomes Secreted by Normoxic and Hypoxic Cardiosphere-derived Cells Have Anti-apoptotic Effect

**Published:** 2018

**Authors:** Helia Namazi, Iman Namazi, Parisa Ghiasi, Hassan Ansari, Sarah Rajabi, Ensiyeh Hajizadeh-Saffar, Nasser Aghdami, Elham Mohit

**Affiliations:** a *Department of Pharmaceutical Biotechnology, School of Pharmacy, Shahid Beheshti University of Medical Sciences, Tehran, Iran. *; b *Students’ Research committee, Shahid Beheshti University of Medical Sciences, Tehran, Iran. *; c *School of medicine, Mashhad university of medical sciences, Mashhad, Iran. *; d *Department of Stem Cells and Developmental Biology at Cell Science Research Center, Royan Institute for Stem Cell Biology and Technology, ACECR, Tehran, Iran.*; e *Protein Technology Research Center, Shahid Beheshti University of Medical Sciences, Tehran, Iran.*

**Keywords:** Cardiosphere-derived cells, Exosomes, Hypoxia preconditioning, Anti-apoptotic effect

## Abstract

Cardiosphere-derived cells (CDCs) have emerged as one of the most promising stem cell types for cardiac protection and repair. Exosomes are required for the regenerative effects of human CDCs and mimic the cardioprotective benefits of CDCs such as anti-apoptotic effect in animal myocardial infarction (MI) models. Here we aimed to investigate the anti-apoptotic effect of the hypoxic and normoxic human CDCs-derived exosomes on induced apoptosis in human embryonic stem cell-derived cardiomyocytes (hESC-CMs). In this study, CDCs were cultured under normoxic (18% O_2_) and hypoxic (1% O_2_) conditions and CDC-exosomes were isolated from conditioned media by differential ultracentrifugation. Cobalt chloride as hypoxia-mimetic agents at a high concentration was used to induce apoptosis in hESC-CMs. The caspase-3/7 activity was determined in apoptosis-induced hESC-CMs. The results indicated that the caspase-positive hESC-CMs were significantly decreased from 30.63 ± 1.44% (normalized against untreated cardiomyocytes) to 1.65 ± 0.1 and 1.1 ± 1.09 in the presence of normoxic exosomes (N-exo) at concentration of 25 and 50 μg/mL, respectively. Furthermore, hypoxic exosomes (H-exo) at concentration of 25 and 50 μg/mL led to 8.75 and 12.86 % reduction in caspase-positive cells, respectively. The anti-apoptotic activity of N-exo at the concentrations of 25 and 50 μg/mL was significantly higher than H-exo. These results could provide insights into optimal preparation of CDCs which would greatly influence the anti-apoptotic effect of CDC-exosomes. Totally, CDC-secreted exosomes have the potential to increase the survival of cardiomyocytes by inhibiting apoptosis. Therefore, CDC-exosomes can be developed as therapeutic strategy in ischemic cardiac disease.

## Introduction

Cardiovascular disease is one of the leading pathological causes of mortality worldwide (1). Cardiosphere-derived cells (CDCs) currently are in phase 2 clinical trials to reverse post- myocardial infarction (MI) injury. The results of studies in various animal models and also a phase 1 human study have shown that the CDCs have the ability to decrease scar mass, increase viable mass, and halt adverse remodeling (2). It was demonstrated that exosomes secreted by CDCs replicate the cardioprotective and regenerative effects of CDCs, including anti-apoptotic effect (3). 

Exosomes, 30-150 nm in diameter lipid bilayer vesicles, are secreted by many cell types and contain a wide range of functional proteins, mRNAs, and miRNA. They are the key transporters of paracrine factors and are able to mediate cell–cell communication. (4). Exosomes as therapeutic agent for repairing damaged myocardium could overcome many obstacles associated with stem cell-based therapy (3, 5).

Immediately after coronary arterial occlusion in acute myocardial infarction, cardiomyocytes are stressed by hypoxia which leads to undergo apoptosis (6). Hypoxia-inducible factor-1 (HIF-1) is the master regulator of the cellular adaption to hypoxic stress (7). 

Recent reports suggested that hypoxia as an *in-vitro* environmental stressors can modify the composition of cardiac progenitor cell-derived exosomes (CPC-exo). These studies concluded that hypoxia have beneficial effect on the cardiac response through paracrine signaling (8, 9).

In the present study, we aimed to investigate the anti-apoptotic effect of CDCs exosomes in cardiomyocytes protection against CoCl_2_-induced apoptosis. We assessed the anti-apoptotic effect of the isolated exosomes from the media of hypoxia (1% O_2_)- and normoxia- treated CDCs on human embryonic stem cell-derived cardiomyocytes (hESC-CMs) in terms of caspase-3/7 activation.

## Experimental

*Cell culture*

Human CDCs (obtained from Iranian pediatric patients diagnosed with a congenital heart disease) were provided by Royan Cell Bank Services. The Patientsꞌ parents gave their informed consent for study participation and research use. Ethical approval was granted by the Royan institute Ethical Committee (10). CDCs were then cultured in proliferation medium [Iscoveꞌs Modified Dulbeccoꞌs Medium (IMDM), Sigma, USA] supplemented with 1% L-glutamine (Invitrogen, USA), 1% penicillin/streptomycin (Invitrogen, USA), 10% fetal bovine serum (FBS, Gibco, USA) with 10 ng/mL basic fibroblast growth factor (bFGF, Royan Biotech) at 37 ºC and 5% CO_2_ in 95% humidity. 

*Cardiac differentiation in static suspension culture*

For hESC-CM production in static suspension culture, 5-day-old hESC spheroids with the size of 175 ± 25 µm were transferred to 60 mm nonadhesive bacterial plates (Sigma-Aldrich, USA) in 5 mL of differentiation medium (DM) which contains Roswell Park Memorial Institute (RPMI) 1640 medium (Gibco, USA) supplemented with 2% B27 without retinoic acid (Gibco, USA), 0.1 mM β-mercaptoethanol, 2 mM L-glutamine, 1% nonessential amino acid. First, the aggregates were treated for one day with 12 μM CHIR99021, a glycogen synthase kinase 3-β inhibitor. Next, spheroids were washed with Dulbecco’s phosphate-buffered saline (DPBS) and were transferred to DM without CHIR99021 for one day. In the next step, the spheroids were transferred to DM containing 5-µM purmorphamine (Stemgent, USA) as the sonic hedgehog agonist, 5 µM IWP2 (Tocris Bioscience, UK) as a Wnt antagonist, 5 µM SB431542 (Sigma-Aldrich, USA) as the inhibitor of transforming growth factor beta (TGF-β) super family type I activin receptor-like kinase receptors. The aggregates were maintained in the medium for two days. On day five, the medium was changed and the spheres transferred to DM after they had been washed with DPBS. The medium was changed every two days. On the seventh day, beating started and reached its highest on the tenth day.

*Exosome purification*

Exosomes were removed from FBS by ultracentrifugation at 120,000 ×g (Type 45 Ti rotor, 32128 rpm, k-factor 133, L-100XP ultracentrifuge, Beckman Coulter, USA) for 18 hours. After discarding the pellet, the supernatant of FBS was filtered through 0.2 µm filters (Techno Plastic Products, Switzerland) and then used in cell cultures (11). Then, CDCs at the fifth passage were cultured in complete media containing IMDM, 10% exosome-depleted FBS, 1% penicillin-streptomycin, and 1% L-glutamine under normoxic (18% O_2_, 5% CO_2_) and hypoxic (1% O_2_, 5% CO_2_ and 94% N_2_) conditions in two distinct incubator (Labotec C200, Germany). The conditioned media was collected 48 h later, and then the exosomes were harvested by differential ultracentrifugation (12, 13). The purified exosome pellet was resuspended in 200 μL PBS and stored at -80 °C. The protein content of the exosome suspension was analyzed by Pierce™ BCA Protein Assay kit (Thermo Scientific, USA). The size of exosomes was determined by dynamic light scattering (DLS) with a Zetasizer nanoseries instrument (Malvern Nano-Zetasizer, UK). The morphological characteristics of exosomes were observed under scanning electron microscopy (SEM, KYKY-EM3200, USA) and flow cytometry was used to analyze surface protein markers of exosomes (detailed explanation of these methods and their results will be reported elsewhere).

*Immunostaining*

hESC-CMs were obtained using a protocol described previously (14). To confirm the differentiation of hESC-CM, cardiac specific markers were stained. To achieve dissociated single cardiomyocytes, the beating spheroids at day 14 of differentiation were washed and maintained in DPBS for 5 min. Then, Accumax cell dissociation solution (Sigma, USA) was added and incubated for 10 min. After that, 5×10^4^ cells/well of the single cardiomyocytes were seeded into 4-well matrigel-coated plates contained fresh DM. Two days later, after washing with DPBS, the attached cells were fixed with 4% (w/v) paraformaldehyde for 20 min at 4 °C and washed with PBS/0.1% Tween 20. Then, the cells were permeabilized with 0.5% Triton X-100 in DPBS for 30-45 min at room temperature. Blocking was performed with 5% (v/v) goat serum for 1h. Next, the cells were incubated with diluted primary antibodies (1:100) in blocking buffer overnight at 4 °C. Primary antibodies used were antibodies against cardiac specific markers: cardiac troponin T (cTnT, Abcam, UK), myosin light chain 2v (MLC2v, Santa Cruz, USA), actinin (Sigma, USA). After three rounds of washing with PBS/0.1% Tween 20 for 5 min each time, the cells were incubated with secondary antibodies [Alexa Fluor 488 goat anti-mouse IgG antibody (Abcam, UK) Alexa Fluor 546 goat anti-mouse IgG antibody (Abcam, UK)] at a dilution of 1:500 in blocking buffer for 45 min at room temperature. Finally, the cardiomyocytes were washed with PBS/0.1% Tween 20 three times. 4ʹ, 6-diamidino-2-phenylindole (DAPI) was used to stain the nuclei for 5-10 min at room temperature. The cells were examined using fluorescence microscopy (Olympus, IX71, USA). The positive hESC-CMS for cardiac specific markers were counted manually in at least five images from different areas of each sample in three independent experiments.

*Apoptosis induction*

Cobalt chloride (CoCl_2_, Sigma, USA) was used for induction of apoptosis in hESC-CMs. The appropriate concentration of CoCl_2_ in order to induce apoptosis in the hESC-CMs was determined. In brief, hESC-CMs were seeded on 3 cm^2^ matrigel-coated plates. Two days later, the cells were treated with different concentrations of CoCl_2_ (1, 2 and 3 mM) for 3 h (3, 15, 16). Then, the caspase-3/7 activity was measured using CellEvent^®^ Caspase-3/7 Green Ready Probes^®^ Reagent (Life technologies, USA) according to manufacturer’s instructions. The samples were analyzed by the flow cytometer (FACS Calibur; BD Biosciences, USA) using Flowing software, version 2.5.1 (BD Biosciences, USA).

*In-vitro apoptosis assay*

In order to determine the anti-apoptotic effect of exosomes, hESC-CMs were cultured on 3 cm^2^ matrigel-coated plates. 24 h later, the cells were treated with 10, 25 and 50 μg/mL normoxic as well as hypoxic exosomes. Apoptosis was induced in hESC-CM by addition of a selected CoCl_2_ concentration after 24 h. The activity of caspase-3/7 was measured 3 h later as described earlier. Apoptosis-induced hESC-CMs with no treatment were used as the positive control of apoptosis induction. 

*Statistical Analysis*

GraphPad Prism software (version 6, USA) was used for statistical analyses. Two independent groups were compared using unpaired student’s T-test. One-way ANOVA followed by Tukey post-test was used to perform multiple group comparisons. The differences with a *p *<0.05 were determined to be statistically significant.

## Results

*CDCs-derived exosome isolation and characterization*

CDCs were cultured under hypoxic or normoxic conditions and the exosomes were isolated from conditioned media by ultracentrifugation after 48 h. The DLS analysis was used to define the size of these exosomes. The mean hydrodynamic diameter of exosomes was between 150–170 nm. Under SEM, the exosomes exhibited a round morphology. The flow cytometry analysis of N-exo and H-exo showed that CD63 and CD81 which are typical exosomal markers, were expressed on the surface of exosomes (Unpublished data).

**Figure 1:**
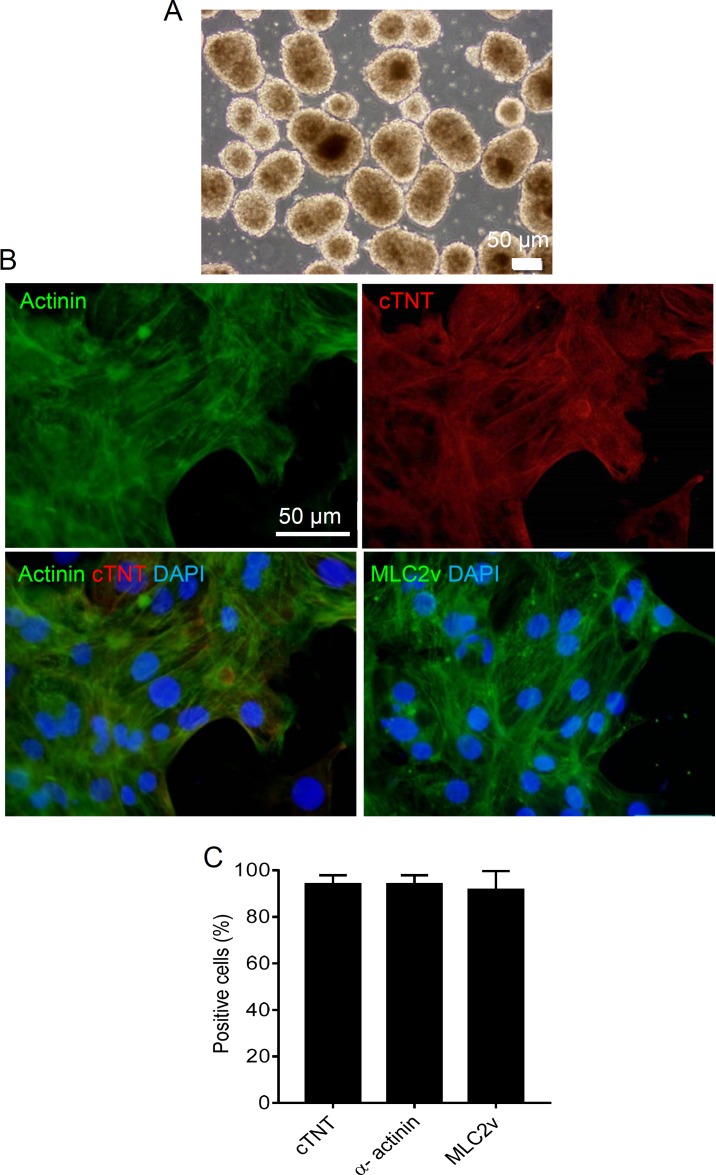
Characterization of hESC-CMs. (A) Beating hESC-CMs spheroids. (B) 14-day-old dissociated beating spheroids were stained for Actinin and MLC2v to show the presence of a sarcomeric structure and the cardiomyocyte-specific structural protein cTnT. Cell nuclei were stained using DAPI. Scale bar: 50 µm. (C) hESC-CMs were >90 % positive for these markers at day 14 of differentiation, Values are mean ± SD

**Figure 2:**
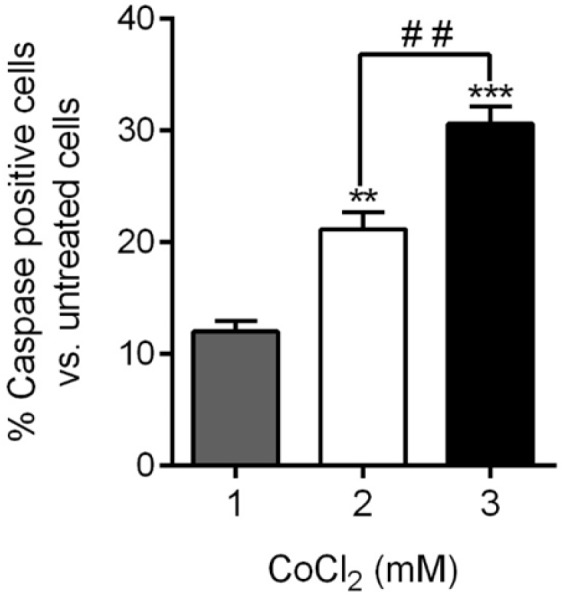
Apoptosis induction. The caspase-3/7 activity was measured to determine apoptogenic concentration of CoCl_2_ on hESC-CMs. Each column represents the mean ± SEM of independent experiments. (**p <0.01, ***p <0.001 vs. 1mM, # # *p <*0.01).

**Figure 3:**
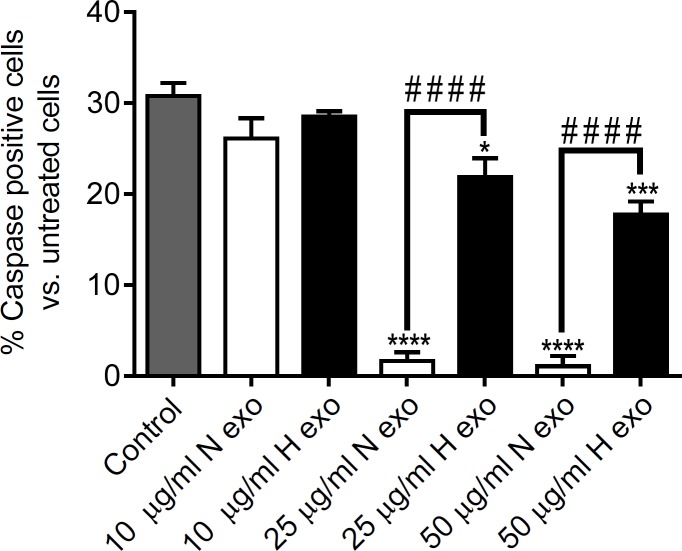
The *in-vitro *anti-apoptotic potential of N-exo and H-exo. The caspase-3/7 activity was measured in CoCl_2_ apoptosis-induced hESC-CMs after treatment with different concentrations of N-exo and H-exo (10, 25 l, 50 μg/mL). N-exo and H-exo inhibited hESC-CMs apoptosis. Each column represents the mean±SEM of three experiments. (* *p*<0.05, *** *p*<0.001, **** *p*<0.0001 vs. control; # # # # *p*<0.0001

*Characterization of hESC-CMs*

The cardiogenic differentiation efficiency was determined by counting the number of beating spheroids at day 10 after the onset of differentiation using an inverted cell culture microscope (Figure 1 A). Approximately 100% of spheroids were beating (see supplementary video online). The 14-day-old beating spheroids were subsequently collected and dissociated for immunostaining of cardiac-specific markers; Actinin, MLC2v and cTnT (Figure 1B). hESC-CMs were approximately 90% positive for the tested cardiac specific markers (Figure 1C).

*CoCl*_2_ *-induced apoptosis in hESC-CMs*

To determine apoptogenic concentration of CoCl_2_ in hESC-CMs, the caspase-3/7 activity was measured in the presence of different concentrations of CoCl_2_ (1, 2 and 3 mM). The analysis showed that treatment of the cells with 3 mM CoCl_2_ for 3 h resulted in 30.63 ± 2.66% cell apoptosis in terms of caspase-3/7 activation. As indicated in Figures. 2, 3 mM CoCl_2_ resulted in significant higher caspase positive cells comparing to than 1 and 2 mM (3 mM vs. 2 mM *p *<0.01, 3 mM vs. 1 mM *p *<0.001).

*The effect of exosomes-derived CDCs under hypoxic and normoxic conditions on induced apoptosis in hESC-CMs*

The effect of hypoxic (H-exo) and normoxic exosomes (N-exo) on CoCl_2_-induced apoptosis was investigated. Accordingly, hESC-CMs were treated with N-exo and H-exo at different concentrations (10, 25 and 50 μg/mL) for 24 h and then the caspase-3/7 activity was measured after apoptosis induction wih CoCl_2_. We found that both N-exo and H-exo inhibited hESC-CMs CoCl_2_-induced apoptosis (Figure. 3). N-exo at concentrations of 25 and 50 μg/mL significantly reduced caspase-positive cells as compared to untreated cells [N-exo (25 and 50 μg/mL) vs. control, *p *<0.0001]. H-exo resulted in significant anti-apoptotic activity at concentration of 25 and 50 μg/mL compared to control [N-exo (25 μg/mL) vs. control, *p *<0.05; N-exo (50 μg/mL) vs. control, *p *<0.001]. Both N-exo and H-exo caused no significant change in anti-apoptotic effects at the concentration of 10 μg/mL (Figure 3). The anti-apoptotic activity of N-exo at concentrations of 25 and 50 μg/mL was significantly higher from that of H-exo at these concentrations [N-exo (25 μg/mL) vs. H-exo (25 μg/mL), *p *<0.0001; N-exo (50 μg/mL) vs. H-exo (50 μg/mL), *p *<0.0001].

## Discussion

CDCs have shown to promote cardiac regeneration of the infracted human heart (2, 17). Exosomes generated by CDCs are beneficial paracrine signals that reproduce CDC-induced therapeutic regeneration. They are sufficient to mediate the entire effect of CDCs (3, 5). Few studies have investigated the therapeutic potential of CDC-exosomes in animal MI models and some other cardiovascular diseases. These studies have shown that exosomes secreted by CDCs replicate the cardioprotective and regenerative effects of CDCs such as apoptosis inhibition of cardiomyocytes. Gallet and coworkers indicated that CDC-derived exosomes delivered by intramyocardial (IM) injection has the ability to decrease acute ischaemia-reperfusion injury, halt adverse remodeling and to improve LVEF in pig models of acute (AMI) and convalescent myocardial infarction (CMI) (2). In the study of Ibrahim *et al.*, exosomes secreted by human CDCs inhibit apoptosis and promote proliferation of cardiomyocytes, while enhancing angiogenesis. Injection of exosomes into injured mouse hearts recapitulates the regenerative and functional effects produced by CDC transplantation, whereas inhibition of exosome production by CDCs blocks those benefits (3). All of these data confirmed *in-vivo* anti-apoptotic effect of CDC-exosomes (2, 3, 18). 

In all of these studies, CDC-exosomes were obtained under normoxic conditions, which likely could not reflect the state of post-infarct tissue. While most *in-vitro* cultured cells maintained at oxygen levels of approximately 20%, natural cell micro-environments seem to have much lower oxygen tensions with considerable variation based on location. For instance the mean oxygen concentration of arterial blood is approximately 12%, and that of tissue is 3% (19). Adult stem cells similarly live under hypoxic conditions of 3-5% O_2_ *in-vivo* and these hypoxic conditions are the physiological norms for a variety of stem cell niches (20). These studies have shown that the level of oxygen play a crucial role in the maintenance, differentiation, and function of stem cells. Nevertheless, hypoxia can also induce mitochondria-mediated apoptosis and subsequent caspases activation in bone marrow-derived mesenchymal stem cells (21). In this study we have analyzed the anti-apoptotic activity of exosomes generated by CDCs under both normoxic and hypoxic conditions. Here, we showed that H-exo and N-exo significantly decrease CoCl_2_-induced apoptosis in hESC-CM.

Here, to isolate the CDC-exosomes from conditioned media, differential ultracentrifugation was used as described in the literatures with some modifications (12, 13). The size of H-exo and N-exo were almost similar, with mean hydrodynamic diameter of 150 to 170 nm. CDC-derived exosomes possessed highly positive expression for exosome surface markers, such as CD63 and CD81 (unpublished data).

CoCl_2_ is a well-established hypoxia-mimicking substance. CoCl_2_-treated cells share common features with cells incubated at 1% oxygen (22). CoCl_2_, as a substrate of the ferrochelatase enzyme, is thought to mimic the hypoxia by binding to the heme molecules (instead of Fe^2+^). It was shown that the expression level of hypoxia-inducible factor-1α (HIF-1α), which is a major transcription factor and key regulator of adaptive responses to hypoxia, is markedly increased following treatment with CoCl_2_ in a dose-dependent manner (23). In this study, CoCl_2_ at concentration of 3 mM was used to induce apoptosis. In the study of Guo *et al.*, U937 and NB4 the cell lines were treated by CoCl_2_ at different concentrations of 150, 200 and 300 μM. They found that at the concentration of 150, 200, and 300 μM the viability is reduced to 55, 20, and 7 % in U937 and to 60, 50, and 25% in NB4, respectively (15). Kim *et al*. also demonstrated that neural cells viability is reached to about 60% after 24 h CoCl_2_ treatment at concentration of 1 mM (16). However, it should be taken into consideration that the method of apoptosis detection is not the same in all studies. For example, trypan-blue exclusion and MTT assay were used to evaluate cell viability by Guo (15) and Kim (16) *et al.*, respectively. Totally, our data are in agreement with the results of Guo *et al.* (15) which indicate CoCl_2_ at the concentration of greater than 50 μM induce apoptosis via mitochondria pathway-mediated caspase-3 activation. (15). 

In this study, we found that exosomes secreted by human CDCs were cultured 48 h under hypoxic and normoxic conditions (1% O_2_) inhibited apoptosis at both 25 and 50 μg/mL concentrations. However, the anti-apoptotic effect of N-exo was significantly higher than that of H-exo at concentration of 25 and 50 μg/mL (*p *<0.0001). In the present study, higher oxygen percentage (1% O_2_) was used for hypoxic preconditioning compared to hypoxic culture condition (0.5% O_2_) that was used in the study of Chacko *et al.* (24). Their results indicated that exposure to sub-lethal hypoxia (0.5% O_2_) for as long as 72 h by itself does not induce cell death by apoptosis in mesenchymal stem cells (MSCs) (24). Furthermore, exposure of hypoxic preconditioned MSCs (HPC-MSCs) (0.5% O_2_, 24 h) to severe hypoxia (0.1% O_2_, 48 h) resulted in signiﬁcant decrease in apoptosis compared with non-HPC MSCs (24). In another study, to imitate myocardial ischemic–reperfusion, isolated MSC were induced by hypoxia/reoxygenation (H/R) [6 h hypoxia (<0.5% O_2_) followed by 12 h reoxygenation (21% O_2_)]. It was found that hypoxia preconditioning (8% O_2_ hypoxia and 30 min of reoxygenation) inhibits H/R-induced apoptosis of MSC in a time-dependent manner (25). Khatibi *et al*. reported that preconditioning with H_2_O_2_ along with CoCl_2_ can cause inhibitory effect on cell apoptosis (26).

Recently, a study has reported that the CPC-derived exosomes might be crucial to protect the cardiomyocytes from apoptosis caused by oxidative stress. In their study, exosomes secreted by mice CPCs which were stressed by 100 μM H_2_O_2_ for 6h (H_2_O_2_-exosomes) decreased the percentage of the apoptotic cells to 13.58%, compared with the 33.29% in H_2_O_2_ group, (cardiomyocytes were stressed by 100 μM H_2_O_2_ for 6 h), whereas the normal exosomes (non-H_2_O_2_ induced) could only reduce the apoptotic percentage to 17.39% (27).

Totally, it can be concluded that different parameters in hypoxia preconditioning of cardiac stem cells including the percentage of oxygen and the duration of hypoxia play critical roles in their anti-apoptotic effect. Therefore, to obtain the optimum anti-apoptotic effect of cardiac stem cells (CSCs)-derived exosomes, further investigation is highly required to choose the proper manner of hypoxic preconditioning. 

Additionally, the way of apoptosis induction in cardiomyocyte might be important in the anti-apoptotic effect of CSCs-derived exosomes. The severe hypoxic (0.1% O_2_) and reduced serum conditions that led to decreasing cells apoptosis in the study of Chacko *et al.*, (24) may be different from the apoptosis condition that was induced by cobalt chloride (3 mM) in our study_. _In the study of Xiao *et al.*, H_2_O_2_ was used to induce the oxidative stress that originates mainly in mitochondria from reactive oxygen species (ROS) (27, 28). Their results demonstrated that H_2_O_2_-exosomes reduce H_2_O_2_ induced apoptosis. Further investigations dealing with the kind of apoptosis induction will be helpful.

We can get to the conclusion that CDC-secreted exosomes have the potential to prevent apoptosis in cardiomyocytes and they will hopefully provide a promising therapeutic strategy for ischemic cardiac disease. Our results imply the need for further investigation of the effect of hypoxia-preconditioning method of cardiac stem cells on the anti-apoptotic activity of their secreting exosomes. 
